# Genome-wide evolutionary and functional analysis of the Equine Repetitive Element 1: an insertion in the myostatin promoter affects gene expression

**DOI:** 10.1186/s12863-015-0281-1

**Published:** 2015-10-26

**Authors:** Marco Santagostino, Lela Khoriauli, Riccardo Gamba, Margherita Bonuglia, Ori Klipstein, Francesca M. Piras, Francesco Vella, Alessandra Russo, Claudia Badiale, Alice Mazzagatti, Elena Raimondi, Solomon G. Nergadze, Elena Giulotto

**Affiliations:** Dipartimento di Biologia e Biotecnologie “Lazzaro Spallanzani”, Università di Pavia, Via Ferrata 1, 27100 Pavia, Italy; Laboratorio di Genetica Forense Veterinaria, UNIRELAB srl, Via A. Gramsci 70, 20019 Settimo Milanese (MI), Italy

**Keywords:** Horse genome, SINEs, Equids, Myostatin gene expression

## Abstract

**Background:**

In mammals, an important source of genomic variation is insertion polymorphism of retrotransposons. These may acquire a functional role when inserted inside genes or in their proximity. The aim of this work was to carry out a genome wide analysis of ERE1 retrotransposons in the horse and to analyze insertion polymorphism in relation to evolution and function. The effect of an ERE1 insertion in the promoter of the myostatin gene, which is involved in muscle development, was also investigated.

**Results:**

In the horse population, the fraction of ERE1 polymorphic loci is related to the degree of similarity to their consensus sequence. Through the analysis of ERE1 conservation in seven equid species, we established that the level of identity to their consensus is indicative of evolutionary age of insertion. The position of ERE1s relative to genes suggests that some elements have acquired a functional role. Reporter gene assays showed that the ERE1 insertion within the horse myostatin promoter affects gene expression. The frequency of this variant promoter correlates with sport aptitude and racing performance.

**Conclusions:**

Sequence conservation and insertion polymorphism of ERE1 elements are related to the time of their appearance in the horse lineage, therefore, ERE1s are a useful tool for evolutionary and population studies. Our results suggest that the ERE1 insertion at the myostatin locus has been unwittingly selected by breeders to obtain horses with specific racing abilities. Although a complex combination of environmental and genetic factors contributes to athletic performance, breeding schemes may take into account ERE1 insertion polymorphism at the myostatin promoter.

**Electronic supplementary material:**

The online version of this article (doi:10.1186/s12863-015-0281-1) contains supplementary material, which is available to authorized users.

## Background

A large fraction of the genome of mammals is occupied by interspersed repeats that were generated during evolution by the propagation of transposable elements [1–3]. Short INterspersed Elements (SINEs) are non-autonomous retrotransposons that make use of a transposition process in which an RNA intermediate is reverse transcribed and the resulting cDNA is inserted into a new genomic location [4, 5]. Sequence analysis of SINE elements suggested that most of them derive from ancestral tRNAs, but there are examples of 5S- or 7SL-like sequences [6]. These elements are characterized by two internal RNA-polymerase III promoters that make them transcriptionally independent, but their retrotranscription and integration processes are catalyzed by enzymes encoded by autonomous Long INterspesed Elements (LINEs) [4, 5]. The primate Alu family is an example of SINE; Alu repeats are the most abundant transposable elements in the human genome accounting for more than one million copies [7–9]. The majority of human Alu elements are present in all individuals because they were inserted in the genome before the radiation of extant humans; however, some Alu elements, that were integrated recently in the human lineage, are characterized by insertion polymorphism [9–12]. In humans, an inverse correlation between the evolutionary age of Alu subfamilies and the percentage of polymorphic elements was demonstrated: 20–25 % of the elements belonging to the youngest subfamily (AluY) are polymorphic [13].

Because of their abundance and mechanism of origin, transposable elements were considered “junk DNA”, albeit, in a number of examples it was shown that they can acquire a functional role, a process termed “exaptation” [14–17]; in particular, the insertion of transposable elements inside genes or in their proximity may alter gene structure or expression through gene interruption, introduction of promoter sequences or splice sites [18–20]. In some rare cases, transposons are implicated in genetic disease or cancer [21–23].

In the present paper, taking advantage of the published horse genome sequence [24], we carried out a genome wide analysis of the perissodactyl-specific SINE family of Equine Repetitive Elements (ERE) focusing our attention on insertion polymorphism in relation to sequence conservation. ERE retrotransposons derive from tRNA^ser^ and occupy about 4 % of the horse genome [25, 26]; to date, four main ERE subfamilies were identified: ERE1-4 [27, 28]. To our knowledge, before the present study, no data were available on the involvement of horse transposable elements in the modulation of gene expression. The description of a polymorphic ERE1 insertion in the promoter of the myostatin gene [29] prompted us to investigate the possible functional role of this insertion.

Myostatin or growth/differentiation factor 8, a member of the transforming growth factor-β family, is a repressor of muscle growth that regulates myoblast proliferation and differentiation. It has been shown previously that mutations in the myostatin gene can cause muscle hypertrophy in a range of mammals such as mice [30], cattle [31, 32] and sheep [33]. In 2004, Schuelke and collaborators reported the case of an extraordinarily muscular child whose mother appeared muscular, although not to the extent observed in her son, and was a professional athlete [34]. The authors discovered that the boy carried a single base substitution in both copies of the myostatin gene generating a premature termination codon while the mother was heterozygous for the mutation. Particularly relevant in this context is also the “bully” phenotype in whippet racing dogs, which depends on a frameshift mutation causing the production of a truncated protein. Individuals homozygous for the mutation show a double-muscle-phenotype, called “bully”, while heterozygotes display an intermediate phenotype. While heterozygous animals have significantly greater racing ability than wild-type and mutated homozygous dogs, the excessive muscle mass of homozygotes for the mutation is detrimental for performance [35].

In the horse, the myostatin gene, which comprises three exons and two introns, is located on chromosome 18; several sequence variants were identified in this gene and in its flanking regions [29, 36–41]; among these variants the SNP g.66493737C > T, which is contained within the first intron, was associated with regulation of gene expression in Thoroughbred race horses and proposed as the best predictor of optimum racing distance [29, 38, 42]. The same variant was also associated with high values of body weight/withers height ratio, which, in the horse, is considered a good indicator of skeletal muscle mass [43]. Four additional SNPs, located in the regions adjacent to the myostatin gene, have been identified on chromosome 18 and were associated to performance [43–45]. Finally, as mentioned above, the insertion of an ERE1 element within the promoter region of the myostatin gene was described in some Thoroughbreds [37]. Recently, the presence of this insertion has been associated with a different muscle fiber composition [40, 46]. In the present paper we tested whether this insertion affects gene expression, contributes to breed differentiation and is relevant for sport aptitude and racing performance.

## Results and discussion

### Insertion polymorphism of ERE loci in the reference genome

A large body of evidence suggests that the horse genome is in a state of rapid evolution [24, 47–50]. Therefore, we may expect that several transposon insertions may have occurred in the horse lineage in relatively recent evolutionary times.

A preliminary *in silico* analysis of the four ERE subfamilies (ERE1 to ERE4) was carried out. To this purpose, the consensus of each ERE subfamily [27, 28] was used as query for a BLAT search (BLAST-Like Alignment Tool) in the reference sequence of the horse [51, 52], which derives from the assembly of the genomic sequence of the Thoroughbred horse named Twilight [24]. From each ERE subfamily, the 200 loci with the highest identity to their consensus were analyzed in search of empty alleles (i.e., alleles in which the ERE element is not present, ERE−) that may be present in the reference genome, thus identifying heterozygous loci in the genome of Twilight. ERE− alleles were found for 3.5 % of the ERE1, 0.5 % of the ERE2 and none of the ERE3 and ERE4 loci. Since the frequency of insertion polymorphism of transposable elements is related to the age of their insertion in the host genome [11], these results strongly suggest that ERE1s are the elements that were inserted most recently in the horse genome. It must be underlined that, since the reference sequence derives from the genome of a single horse, the frequencies of polymorphic loci reported above are largely underestimated being based on the analysis of two alleles per locus.

We then focused on the youngest subfamily, the ERE1, and carried out an extensive genome wide search of these elements in the reference genome sequence (Broad/equCab2). A list of 45,713 ERE1 loci was obtained using the consensus sequence deposited at the RepBase database as query [53] for a BLAST search (Additional file 1: Table S1A). The sequences were then filtered to include only elements with sizes similar to the ERE1 consensus (225 bp ± 10 bp) and with minimum identity of 84 % to the consensus. This operation left 34,131 loci (Additional file 1: Table S1B). The ERE1 sequences located inside other repetitive elements were also excluded from the analysis to avoid false positive results; this operation left 27,396 loci (Additional file 1: Table S1C). In order to obtain a comprehensive view of polymorphic ERE1 loci in Twilight, we analyzed the horse trace database, which includes unassembled traces [54] (center_project number G836). The sequence of each one of the 27,396 ERE1 loci was used as query for a BLAST search. The results of this analysis showed that Twilight is heterozygous at 377 ERE1 loci, possessing an ERE1+ and an ERE1− allele. A complete list of these polymorphic loci is reported in Additional file 2: Table S2. It is important to point out that an undefined number of ERE1 insertions, that are present in the horse population, is not detectable in the reference genome because Twilight may carry two ERE1− empty alleles at such loci. A clear example of this situation is the insertion in the myostatin gene promoter described below.

**Fig. 1:**
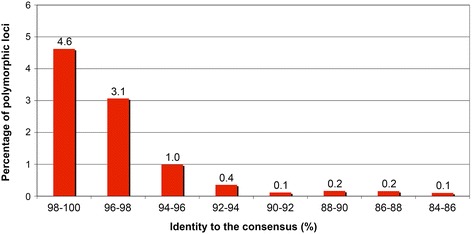
Percentage of ERE1 polymorphic loci in the horse genome reference sequence. The ERE1 elements were grouped in eight classes according to their identity to the ERE1 consensus sequence published in Repbase. The percentage of polymorphic loci in each class is reported

**Fig. 2:**
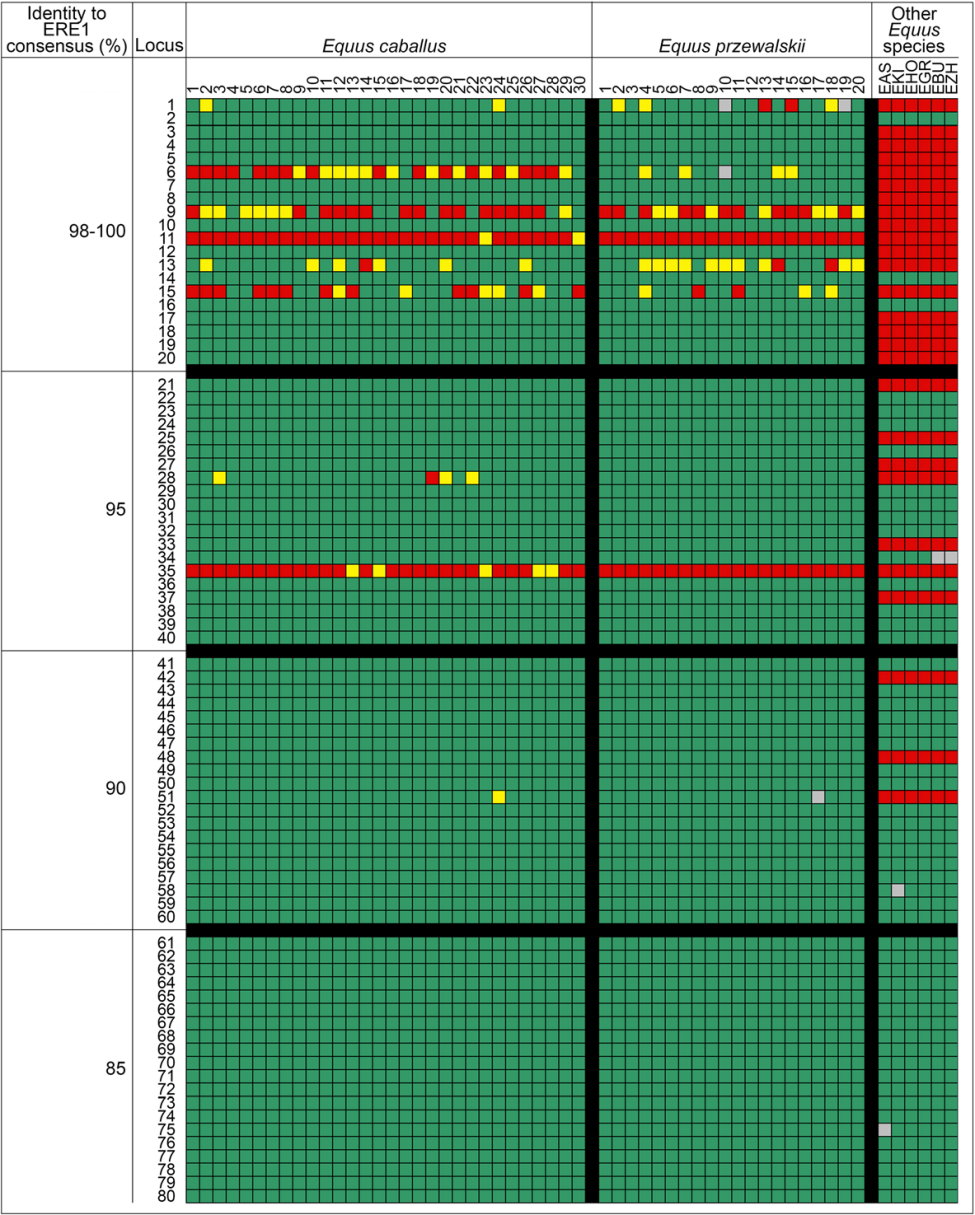
Insertion polymorphism of 80 ERE1 loci in equids. The insertion polymorphism of 80 random ERE1 loci with different percentage of identity to the ERE1 consensus were analysed: 20 loci with 98–100 %, 20 loci with 95 %, 20 loci with 90 % and 20 loci with 85 % identity. The analysis was carried out in 30 individuals from *E. caballus,* 20 individuals from *E. przewalskii*, three individuals from *E. asinus*, EAS, and one individual from each one of the following species: *E. kiang*, EKI; *E. hemionus onager*, EHO; *E. grevyi*, EGR; *E. burchellii*, EBU; *E. zebra hartmannae*, EZH. The position of each locus in the horse genome is reported in the left column. Each column reports data from the animal indicated on top. Each table cell shows the genotype of an individual at a specific locus. Genotypes are indicated using different colours: green, homozygous for the ERE1+ allele; red, homozygous for the ERE1- allele; yellow, heterozygous; grey, no data

Since the fixation of insertion elements in the genome of a phylogenetic lineage requires many generations, the presence of empty alleles suggests that the insertion event occurred in relatively recent evolutionary times. In addition, mutations tend to accumulate in the inserted element and therefore a high degree of sequence conservation is considered indicative of a young evolutionary age of insertion, as previously shown for primate and rodent interstitial telomeric sequences [55, 56] and for human transposable elements [57]. In light of these considerations, we can hypothesize that ERE1 elements with higher identities to the consensus may have greater probabilities of being polymorphic compared to less conserved elements. To test this hypothesis, we evaluated the frequency of polymorphic loci in eight classes of ERE1 elements, characterized by different degrees of identity to the consensus (Fig. 1 and 2; data file 1: Table S3). In the class including ERE1 loci with the highest identity to the consensus (98–100 %), the percentage of loci that are polymorphic in Twilight is surprisingly high (4.6 %); this fraction decreases with the decrease of identity to the consensus reaching values as low as 0.1 % (Fig. 1). The correlation between fraction of polymorphic loci and percentage of identity to the ERE1 consensus sequence is highly significant (Pearson’s correlation *ρ* = 0.93, *p* = 8.5 × 10^−4^). These results suggest that sequence conservation and insertion polymorphism of ERE elements are both related to the time of their appearance in the horse lineage.

### Insertion polymorphism in the horse population, evolutionary history and sequence conservation of ERE1 loci

To evaluate the frequency of insertion polymorphism in the horse population, we analyzed 80 ERE1 loci in 30 unrelated domestic horses of different origin (see Materials and Methods). The 80 loci were chosen randomly from four classes (20 loci per class) with different degrees of identity to the ERE1 consensus sequence (≥98, 95, 90 and 85 % identity). For each locus, a primer pair flanking the ERE1 element was designed (Additional file 2: Table S4) and the genomic DNA of the 30 horses was amplified by PCR. The analysis of these loci in the 30 horses is summarized in Fig. 2, where different colours indicate the genotypes of each individual: ERE1+/+, green; ERE1+/−, yellow; ERE1−/−, red. For 71 loci (Fig. 2) only individuals homozygous for the presence of the ERE1 element (ERE1+/+) were found, suggesting that either the insertion is fixed in the population or the frequency of ERE1- alleles is very low. The remaining 9 loci were characterized by insertion polymorphism (Fig. 2). At these 9 loci, the fraction of ERE1- alleles per locus is highly variable ranging from 1.7 (locus 51) to 97 % (locus 11). Although the number of loci analyzed in each class as well as the number of individuals are relatively small, the results are in agreement with the *in silico* results described above: polymorphic loci are more represented in the class with the highest similarity to the ERE1 consensus sequence (6 loci out 20) whereas no polymorphic loci were identified in the class with the lowest identity to the consensus. These results confirm the observation, reported in the previous paragraph, that elements with high similarity to the consensus sequence, have a greater probability of being polymorphic compared to less conserved elements. We previously observed a high frequency of insertion polymorphism in the horse, involving NUMT elements (NUclear sequences of MiTochondrial origin) [49]. Similarly to NUMT sequences, the fraction of ERE1 polymorphic loci described here is particularly high compared to that reported for SINE elements in the human genome [9], thus providing further evidence for the rapid evolution of the horse genome.

**Fig. 3:**
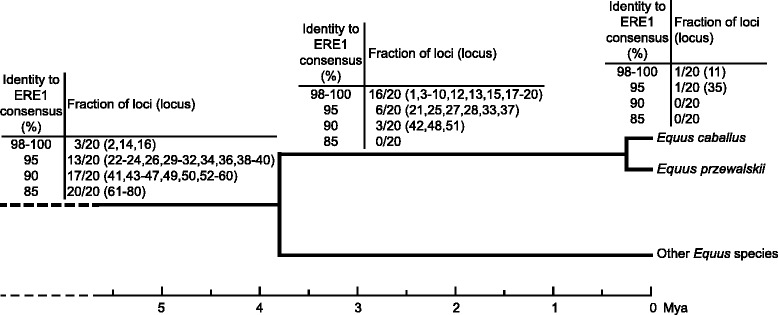
Phylogenetic tree of equids. The time of insertion of each one of the 80 ERE1s is marked on the phylogenetic tree (adapted from [58, 59]). ERE1 loci are classified according to the percentage of identity to the consensus sequence, the fraction of inserted loci in each class of identity is shown. Each ERE1 is indicated by a unique locus number (see Fig. 2 and Additional data file 1: Table S3A). The lineage “Other *Equus* species” comprises the following non-horse species: *E. asinus*, *E. kiang*, *E. hemionus onager*, *E. burchellii*, *E. grevyi*, *E. zebra hartmannae*

We also analyzed the 80 loci in 20 Przewalski’s horses, in three individuals from *E. asinus* and in one individual each from *E. burchellii*, *E.grevyi*, *E. zebra hartmannae*, *E. kiang* and *E. hemionus onager*, respectively (Fig. 2); since the results of the three *E. asinus* individuals were identical, only one column is reported in Fig. 2. As shown in Fig. 3, from the evolutionary point of view, ERE1 loci can be classified in three groups: elements which are conserved in all species of the genus *Equus* (53 loci) and thus were inserted in a common ancestor of all extant equids, at least 3.8 Ma ago (Mya); elements which are conserved in all analyzed horses (*E. caballus* and *E. przewalskii*) but absent in the other *Equus* species (25 loci), thus inserted after the separation of the horse lineage, that is about 3.8 Mya [58, 59]; elements which are present in *E. caballus* only (two loci: 11 and 35 in Fig. 2) and therefore were probably inserted after the separation of the two horse species. To this regard, it must be pointed out that, in the middle of the twentieth century, Przewalski’s horses were close to extinction and the extant population derives from a very limited number of individuals [60]; therefore, the absence of an ERE1 element in Przewalski’s horses may be related either to the date of its insertion or to genetic drift. Nine loci (number 1, 6, 9, 11, 13, 15, 28, 35, 51 in Fig. 2) are polymorphic in one or both horse species and absent in the other species, suggesting that these insertions occurred in a relatively recent evolutionary time, after the separation of the horse lineages, and are not yet fixed.

In conclusion, these results showed that the fraction of ERE1 insertions conserved in all *Equus* species increases with the decrease of their identity to the consensus (Fig. 3): only 3 out of the 20 horse ERE1 elements with 98–100 % identity were present in the other species while 13, 17 and 20 loci out of 20 were conserved in the classes with 95, 90 and 85 % identity, respectively (Fig. 3). On the contrary, the majority of ERE1s that are present in the horse lineage only (16/20) share a high identity to the consensus (98–100 %). The loci that were conserved in all *Equus* species were not polymorphic in the horse (Fig. 2) confirming that they were inserted earlier during evolution, in a common ancestor of the extant *Equus* lineages. Since only three individuals from *E. asinus* and one individual from *E. burchellii*, *E. grevyi*, *E. zebra hartmannae*, *E. kiang* and *E. hemionus onager* were analyzed, we cannot exclude that, at some ERE1 loci, insertion polymorphism may be present in one or more *Equus* species, however, the results confirm that the level of identity to the consensus not only is related to their polymorphism but is also indicative of their evolutionary age. Therefore, ERE1 insertion polymorphism can be used for evolutionary analyses and population studies.

### Position of ERE1 loci relative to genes

**Fig. 4:**
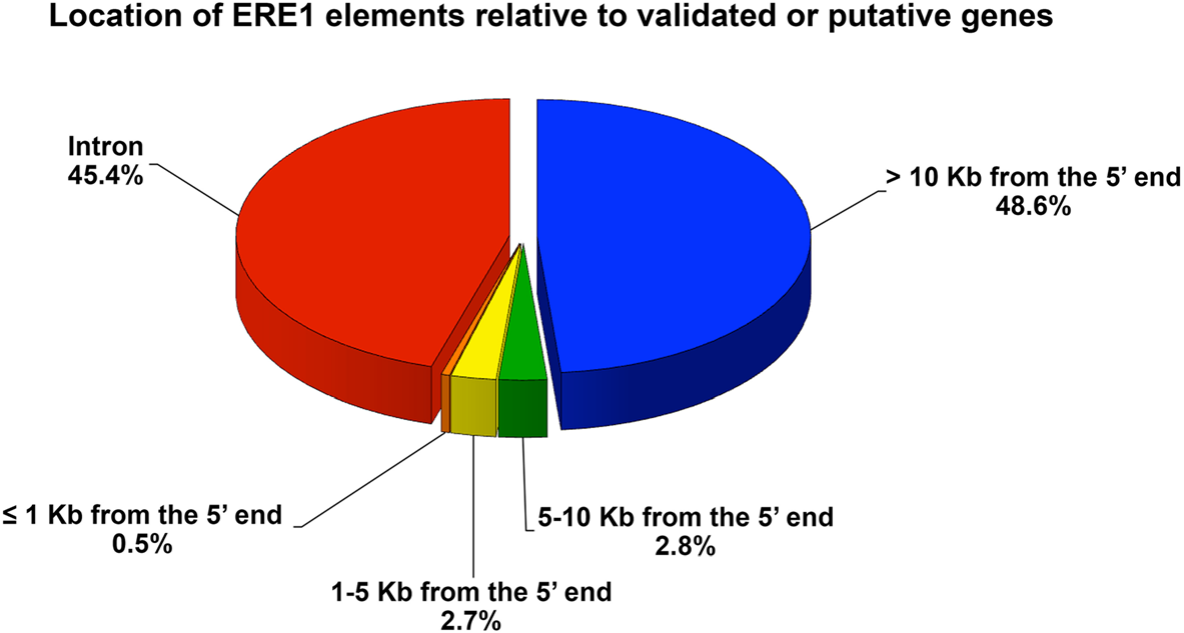
Distribution of ERE1 elements relative to genes. The percentage of ERE1 loci located in introns of validated or putative genes (red) and in non-genic regions is indicated. ERE1 elements located in non-genic regions are classified according to their distance from the 5’ end of the nearest gene (>10 Kb, 5–10 Kb, 1–5 Kb, ≤ 1 Kb)

Since transposable elements, when inserted within or near genes, may influence gene expression, we used an algorithm developed in our laboratory (see Material and Methods) to classify ERE1 elements according to their position relative to genes. The coordinates of the horse genes were obtained using the tool “UCSC Table Browser” [61, 62]. Horse genes are poorly mapped, therefore we included in the analysis the coordinates of putative horse genes listed in a table generated by UCSC, based on homology with human and bovine genes. The results (Fig. 4) showed that 45.4 % of ERE1 elements were located inside introns of validated or putative genes. The fraction of the human genome occupied by introns has been estimated to be between 26 and 38 % [2, 63–67]; since no data are available for the horse, we are unable to conclude whether the fraction of ERE1 elements contained within introns is simply due to random insertion. Given the high number of ERE1 elements within introns, it is possible that some have acquired a functional role by modifying the splicing pattern as documented for other SINEs [68–70]. The remaining ERE1s (54.6 %) were located at variable distances from genes. Our data suggest that there are no hotspots for ERE1 integration sites in the horse genome and that insertion events may have occurred at random. Counter-selection may be responsible for the lack of insertions within exons. Moreover, only 170 ERE1 insertions (0.5 %) were found at less than 1 kb from the 5’ end of validated or putative genes suggesting that some of them may affect gene expression.

### Sequence organization of the myostatin gene promoter and mechanism of ERE1 insertion

**Fig. 5:**
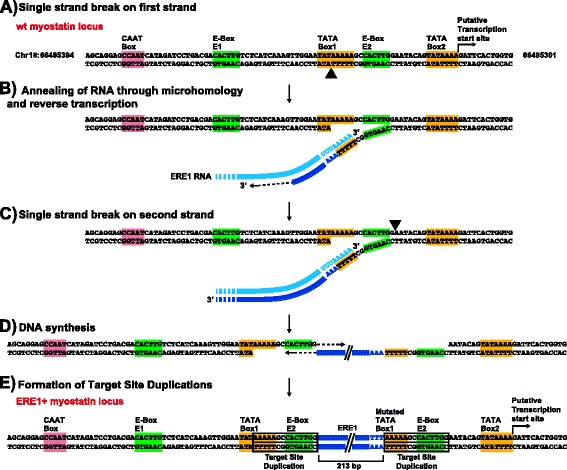
Model for ERE1 integration via retrotransposition at the myostatin promoter. **a** Sequence of the empty wild type myostatin locus, the coordinates of the locus in the horse genome reference sequence (equCab2.0) are indicated on both sides. The promoter elements located upstream the transcription start site are shown: CAAT-box (pink), E-boxes (green), TATA boxes (yellow). The black arrowhead points to the position of the single strand break. **b** Annealing through microhomology (TTTTT/AAAAA) of the ERE1 RNA (light blue) to the single stranded DNA end originated after the nick and reverse transcription. The cDNA produced by retrotranscription is shown in dark blue. **c** Cleavage of the second DNA strand (black arrowhead). **d** Synthesis of the second strand of ERE1 cDNA and gap filling. **e** Sequence of the ERE1+ myostatin locus: the ERE1 element is integrated (dark blue) and the target site is duplicated (boxed nucleotides)

As mentioned above, a polymorphic ERE1 insertion was identified at the myostatin locus [29]. In Fig. 5, the wild type myostatin locus (Fig. 5a), the ERE1+ allele (Fig. 5e), and a model for the transposition mechanism (Fig. 5b–d) are shown. At the wild type myostatin locus, the regulatory elements, located upstream and in close proximity of the putative transcription start site (Fig. 5a), comprise: two TATA boxes (TATA box1 and 2, located 24 and 1 bp upstream the transcription start site, respectively) and one CAAT box (70 bp upstream the transcription start site). In addition, two E-boxes (E1 and E2), which are muscle gene control elements [71, 72], are located 49 and 16 bp upstream the transcription start site, respectively. Given their position relative to the putative transcription start site, the TATA Box 1 and the CAAT box are likely to constitute the core promoter directing transcription of the horse wild type myostatin gene.

Sequence comparison of the wild type and ERE1+ alleles suggested that this insertion may have occurred according to the previously proposed mechanism of SINE elements retrotransposition in the human genome leading to a direct duplication of the target site [16, 73, 74]. According to this model, during the first step of the process (Fig. 5a), the target site was cleaved inside the TATA box 1 (black arrowhead); the 3’ end of the ERE1 RNA (light blue) annealed through microhomology to the single-stranded 5’-TTTTT-3’ sequence generated after the nick in the TATA box 1 (Fig. 5b). The free 3’OH group created after the cleavage was then used to prime the reverse transcription of the ERE1 RNA and synthesize the first strand of the cDNA (dark blue, Fig. 5b). The second strand of the DNA was then cleaved one bp downstream the E-box E2 (black arrowhead, Fig. 5c), producing a 3’ end that was used to prime the synthesis of the second strand of the ERE1 DNA (Fig. 5d). Through a gap filling reaction, the entire ERE1 sequence was integrated into the myostatin promoter with the formation of the Target Site Duplication. Fig. 5e shows the ERE1+ allele of the myostatin promoter obtained as a result of the retrotransposition event. The inserted ERE1 (dark blue) is located 29 bp upstream the transcription start site. The size of the Target Site Duplication (14 bp) falls into the range described for SINE elements in the human genome [16, 73, 74]. The consequence of the ERE1 insertion was a modification of the core promoter with the formation of a variant TATA Box 1 and the displacement of the CAAT box. This rearrangement likely affects the strength of the core promoter.

### Reporter gene assay of the two variants of the myostatin gene promoter

To test the hypothesis that the ERE1 insertion alters the expression of the myostatin gene, we performed a reporter gene assay using a plasmid containing the enhanced Green Fluorescent Protein (*eGFP*) gene and the puromycin resistance gene. The two variants of the myostatin promoter (ERE1+ and ERE1-) were cloned from the genomic DNA of a heterozygous Thoroughbred horse and inserted into the plasmid cloning site upstream of the *eGFP* reporter gene. The ERE1- variant plasmid contained a 2042 bp genomic fragment comprising 31 bp from the myostatin UTR; the ERE1+ plasmid contained an insert differing from the previous one only for the ERE1 insertion.

**Fig. 6:**
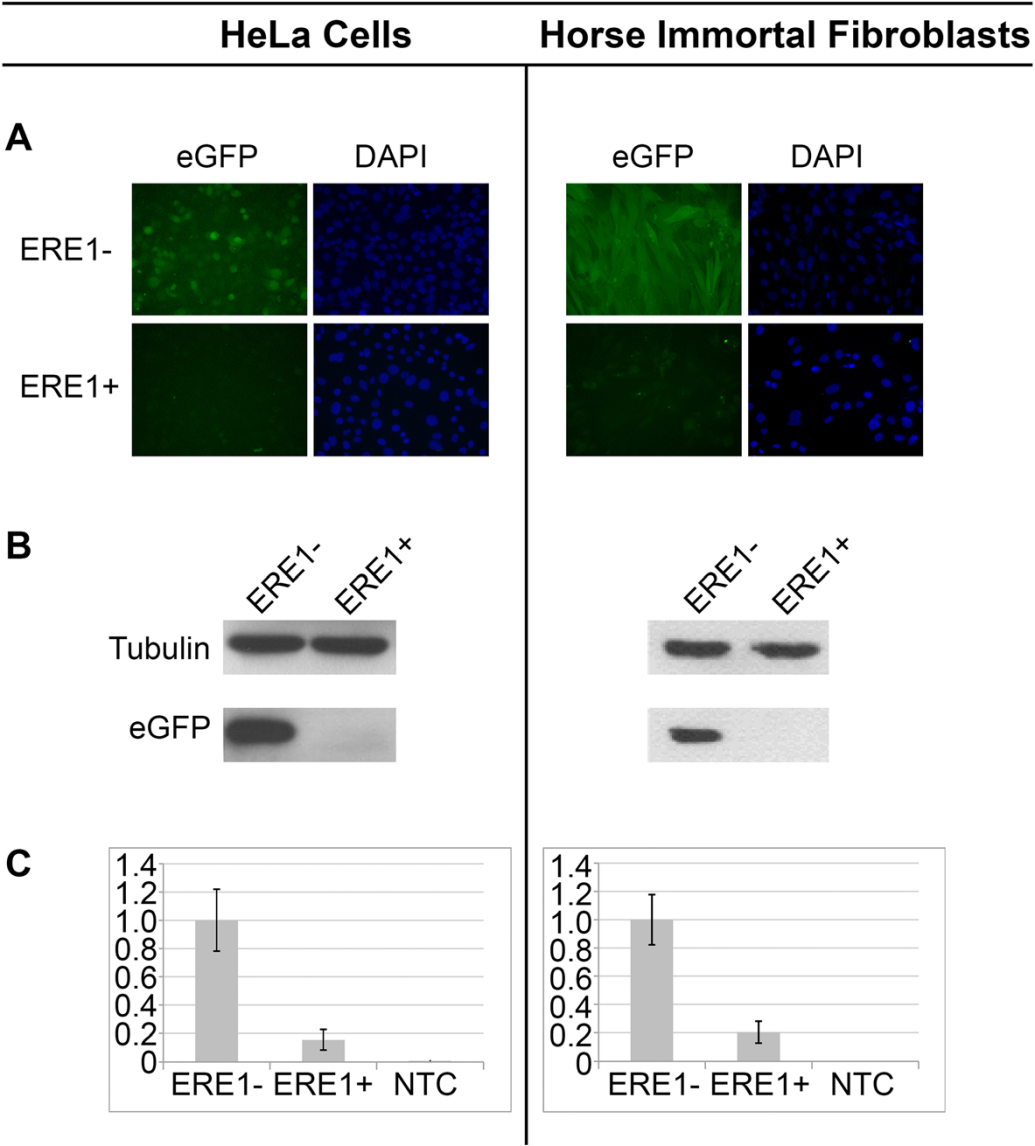
Reporter gene assay. The reporter gene assay (*eGFP* expression) was carried out in human HeLa cells (left) and in horse immortalized fibroblasts (right). **a** Fluorescence microscopy images of cells transfected with the two constructs containing the *eGFP* gene under the control of the ERE1- (top) and ERE1+ (bottom) promoter. DAPI-staineing is shown on the right of each image. **b** Western blots with anti-tubulin (loading control, top) and anti-eGFP (bottom) antibodies using protein extracts from cells transfected with the plasmid containing the ERE1- (left) and the ERE1+ (right) promoter. **c** Quantification of *eGFP* expression by quantitative RT-PCR. The expression levels of the eGFP transcript are indicated in arbitrary units. eGFP levels in cells transfected with the ERE1- plasmid were used as reference and set to 1.0. NTCs, no-template controls

To test whether the ERE1 insertion can affect promoter strength the two plasmids were transfected in human HeLa cells and in a horse fibroblast cell line that we immortalized using the procedure described in Vidale et al. [75]. Since transfection efficiency in horse fibroblasts is extremely low (3–5 %), transient short term transfections could not be performed. Long-term selection with puromycin had to be carried out in order to isolate stably transfected cell populations. The expression of *eGFP* was evaluated by fluorescence microscopy, western blotting and quantitative real-time PCR (Fig. 6). Both in human and in horse cells, the ERE1 insertion caused a reduction of eGFP fluorescence signals to almost undetectable levels (Fig. 6a). The effect of the insertion on promoter strength was also demonstrated by immunoblotting of protein extracts with an anti-eGFP antibody (Fig. 6b): while a strong band could be detected in protein extracts from cells transfected with the plasmid containing the ERE1- promoter, only a very faint band could be observed in extracts from cells transfected with the ERE1+ plasmid. We then carried out a quantitative real-time PCR reaction using eGFP specific primers (Additional file 2: Table S4B) to amplify reverse transcribed mRNA from the transfected cell lines (Fig. 6c): in human cells transfected with the ERE1+ plasmid the expression level of the reporter gene showed a 6.4-fold reduction compared with that observed in cells transfected with the vector carrying the ERE1- promoter; similarly, a 4.9-fold reduction was observed in horse fibroblasts. These results demonstrate that the ERE1 insertion affects the ability of the myostatin gene promoter to drive transcription of a reporter gene and strongly suggest that the myostatin gene may be under-expressed in horses containing this variant promoter sequence.

### ERE1 insertion polymorphism at the myostatin locus: sport aptitude and racing performance

Given the role of myostatin in the regulation of muscle development and considering the relevance of muscular mass in athletic performance, we wondered whether the genotype of horses relative to the ERE1 insertion may influence their sport aptitude and racing abilities.

Using primers flanking the myostatin gene promoter (Additional file 2: Table S4B), we set up a PCR assay to identify the two alleles: the ERE1 containing allele, ERE1+, produces a 441 bp band, while the allele lacking the insertion, ERE1-, produces a 214 bp band. We then analyzed the frequency of the two alleles, in 5 horse breeds (Quarter Horse, Andalusian, Lipizzaner, Norwegian Fjord and Icelandic Pony) and in Przewalski’s horse. As shown in Table 1A, in Quarter horses, although the number of individuals analyzed is limited (20), the frequency of the ERE1+ allele seems particularly high (57 %). In the Andalusian breed, the ERE1+ allele was observed only in 3 heterozygous individuals, while in the other breeds and in Przwelaski’s horse the ERE1+ variant was not present. Since the ERE1 insertion was present only in horse populations in which Thoroughbred blood is known to have been introduced (Quarters, Andalusians, Show Jumpers), it is likely that it appeared recently in the horse lineage and probably occurred in a Thoroughbred ancestor, as previously suggested [46].

**Table 1:** ERE1+ and ERE1- genotyping at the myostatin locus

			Number of alleles (%)		Homozygous individuals (%)		Heterozygous individuals (%)
		Number of individuals	ERE1+	ERE1-	ERE1+/+	ERE1−/−	ERE1+/−
A	Quarter Horse	20	23 (57.5)	17 (42.5)	9 (45)	6 (30)	5 (25)
	Andalusian	20	3 (7.5)	37 (92.5)	0	17 (85)	3 (15)
	Lipizzaner	23	0	46 (100)	0	23 (100)	0
	Norwegian Fjord	20	0	40 (100)	0	20 (100)	0
	Icelandic Pony	19	0	38 (100)	0	19 (100)	0
	Przewalski’s Horse	20	0	40 (100)	0	20 (100)	0
B	Show Jumpers	30	1 (1.7)	59 (98.3)	0	29 (96.7)	1 (3.3)
	Italian Trotters	90	0	180 (100)	0	90 (100)	0
	Unselected Italian Thoroughbreds	75	65 (43.3)	85 (56.7)	18 (24.0)	28 (37.3)	29 (38.7)
	Elite Italian Thoroughbreds	117	135 (57.7)	99 (42.3)	33 (28.2)	15 (12.8)	69 (59.0)

(A) Analysis of individuals from five breeds of the domestic horse and from Przewalski’s horse. (B) Analysis of individuals bred for different sport aptitude.

Although the number of individuals tested for each breed is relatively small (19–23 animals per breed), the striking frequency variation of the two alleles suggests that the two variants may have been under selection during the establishment and improvement of some breeds in relation to specific aptitude and performance traits. In particular, the high frequency of ERE1+ alleles in Quarter horses suggests that this variant may favor the ability of sprinting short distances. To this regard, it is important to point out that the name of this breed came from its excellence in races of a quarter mile or less.

Therefore, to test the hypothesis that the ERE1 insertion at the myostatin locus may affect the aptitude for specific sport abilities, we initially analyzed the frequency of the two allelic variants in 30 horses competing in show-jumping at various levels, in 90 horses registered in the Italian Trotter studbook, bred for harness racing, and in 75 horses registered in the Italian Thoroughbred studbook mainly bred for flat racing (Table 1B). Although Italian Trotters derive from English Thoroughbred stallions crossed with mares of different origins, and Thoroughbreds have been introduced in several bloodlines of Show Jumpers, the allelic frequencies in the three groups were strikingly different (Table 1B): the ERE1+ allele was completely absent in the Trotters and, in the Show Jumpers, only one individual was heterozygous for the variant; on the contrary, among the flat racing horses, the percentage of ERE1+ alleles was 43. These observations suggest that the ERE1+ allele may have been selected in the Thoroughbreds and in the Quarter Horses together with flat racing aptitude.

**Fig. 7:**
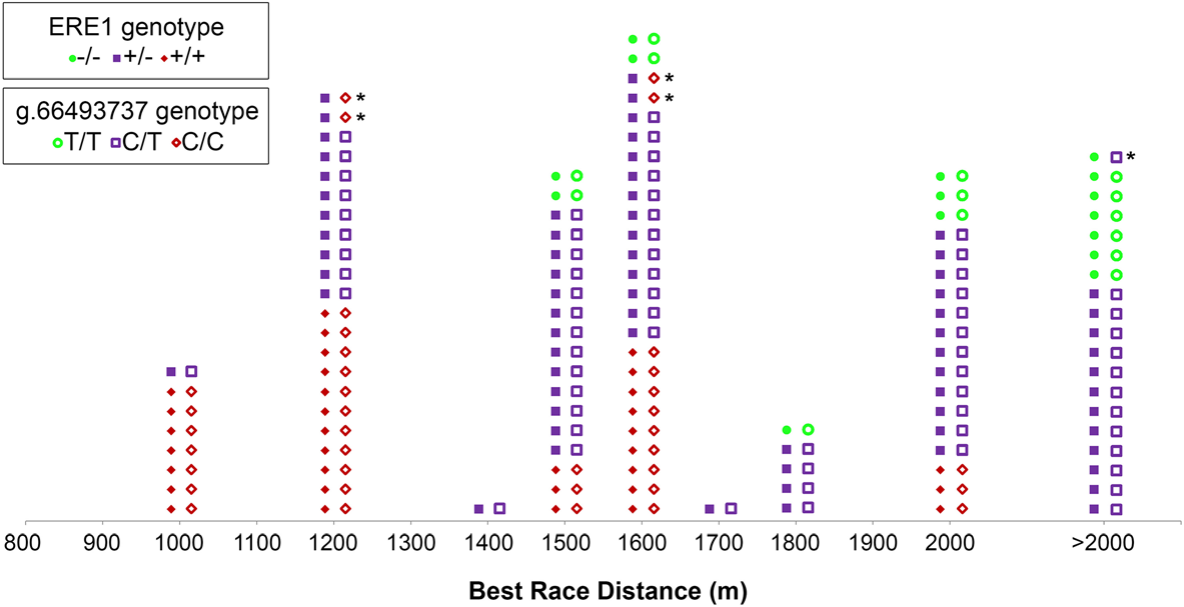
Genotyping of elite Thoroughbred horses. Elite Thoroughbreds (*n* = 117) were classified according to the distance of the highest grade race won (Best Race Distance), expressed in meters. For each individual, the myostatin promoter (full symbols) and the SNP g.66493737C > T (empty symbols) genotypes are shown. The majority of the ERE1−/−, ERE1+/− and ERE1+/+ individuals have a T/T, C/T and C/C SNP genotype, respectively; the five individulas with a different combination of genotypes at the two loci are marked with an asterisk

To test whether the ERE1+ variant may influence racing performance in the Thoroughbreds, we selected a group of 117 elite horses classified in the top three places in at least one high level race in Italy in the period ranging from 2005 to 2011. In this selected group, the ERE1+ allele was significantly more frequent compared to the general Thoroughbred population (*p* = 9.31 × 10^−**6**^, Table 1 B). To test whether the ERE1 insertion influences performance relatively to race distance, the elite horses were grouped according to Best Race Distance, defined as the distance of the highest grade race won. When multiple races of the same grade were won, the distance of the race with the most valuable prize was considered. The results of this analysis are shown in Fig. 7: in short distance races (1000 and 1200 m), the majority of winning horses (18 out of 30) were homozygous for the ERE1+ allele and no homozygous individuals for the ERE1- allele were found; in the long distance races (>2000 m), only heterozygotes and ERE1- homozygotes were observed and, in medium distance races (1400–2000 m), all the three genotypes were represented although the ERE1+ homozygotes were relatively more frequent in the groups winning up to 1600 m races compared to horses winning 1700–2000 m races. When the genotypic frequencies in horses winning short distance (1000–1200 m), medium distance (1400–2000 m) and long distance (>2000 m) races were compared, the differences were highly significant (*p* = 1.94 × 10^−6^).

Since the ERE1+ variant is associated with better performance in short distance races, it may have been artificially selected through breeding, consequently, its frequency increased in the Thoroughbred population, although it was not fixed. The empty allele might also have been subjected to artificial selection. Thoroughbreds are also used for long distance races, in which individuals homozygous for the ERE1- alleles have the best performance while heterozygous animals seem to be advantaged in average distance races. It should be pointed out that among the Italian Trotters, a breed derived from English Thoroughbreds, no ERE1+ allele was identified. This is probably due to the fact that Italian Trotters are bred for harness racing at a trot gait in relatively long distance races and this artificial selection led to the loss of the ERE1+ allele. Finally, although Quarter Horses derive from the crossing of Thoroughbreds with horses from other breeds, the frequency of the ERE1+ allele was even higher than in the Thoroughbreds themselves (Table 1); this observation can be related to the fact that these horses have been selected for their sprinting ability in flat races of a quarter mile or less.

As mentioned in the introduction, the g.66493737C > T SNP in the first intron of the myostatin gene was shown to be predictive of athletic performance [29, 37]: C/C horses are suited for short-distance, C/T for middle-distance and T/T for long-distance races. Comparing the ERE1 and the g.66493737C > T genotypes (Fig. 7), we observed that in 112 out of 117 horses the two genotypes were concordant, with the C SNP allele associated with ERE1+ and the T SNP allele associated with the ERE1- promoter. These results show that the two polymorphic loci are tightly linked, as expected by their close proximity in the genome (1605 bp). Although the ERE1 insertion was previously described [37], its influence on myostatin gene expression was not investigated. In the present work, we demonstrate that the ERE1 insertion affects gene expression supporting the hypothesis that this is the genotype that drove selection [46]. In particular, we showed that the ERE1 insertion causes a 5–6 fold decrease in the transcription of the reporter gene (Fig. 6), providing the first example of a SINE element influencing gene expression in the horse genome.

Although the g.66493737C > T SNP showed an association with racing performance [29], this sequence variation does not provide an immediate functional explanation of this trait. On the contrary, our experimental data strongly suggest a direct influence of the ERE1 insertion on myostatin expression. Since the g.66493737C > T SNP is located only 1605 bp away from the ERE1 insertion site in the promoter, the ERE1 insertion, rather than the g.66493737C > T SNP (located in the first intron), may functionally influence racing performance, the two polymorphisms being in linkage disequilibrium (r^2^ = 0.73) as previously observed [29, 46]. In other words, the results presented here on myostatin expression provide a physiological interpretation of the correlation between ERE1 insertion and racing performance; moreover, the previously described correlation among the g.66493737C > T SNP, muscle mass [43] and muscle fiber composition [46] can also be reinterpreted on the basis of the linkage disequilibrium between the two polymorphic loci.

## Conclusions

In the work presented here we provide a catalogue of the most abundant SINE retrotransposons, ERE1, in the horse genome. Through the analysis of sequence conservation, insertion polymorphism and presence in other equids, we provide an evolutionary dating of ERE1 elements appearance in the *Equus* lineage. Therefore, similarly to other mammalian SINE elements, ERE1 insertion polymorphism can be used for evolutionary analyses and population studies.

The analysis of ERE1s position relative to genes suggests that some may have acquired a functional role by modifying the splicing pattern, when interrupting an intron, or by altering gene expression, when inserted inside regulatory regions. To this regard, we studied the effect of an ERE1 insertion in the promoter of the myostatin gene showing that it causes a reduction of promoter strength in a reporter gene assay. Therefore, we suggest that this ERE1 insertion may decrease the levels of myostatin thus modifying muscle development.

The ERE1 insertion at the myostatin locus is polymorphic in the horse population and seems to be related to specific racing aptitude, the ERE1+ allele being particularly common in breeds characterized by sprinting ability, such as the Quarter Horse, and absent in other breeds, such as the Italian Trotter, which are used for long distance racing. In a sample of Thoroughbred elite horses, classified in the top three places in at least one high level race in Italy, we observed a statistically significant correlation between the ERE1+ variant and good performance in short distance races; on the other hand, the empty allele was more frequent in Thoroughbreds winning long distance races. We propose that the two variants have been unwittingly selected by breeders in order to obtain horses with specific racing abilities. Our results indicate that, although racing performance is certainly influenced by environmental factors, like training and nutrition, and by several genetic factors, breeding schemes may also take into account the differential effect of these two ERE1 allelic variants.

## Methods

### Ethics statement

Horse blood and hair samples were collected in the stables where the animals were kept, during veterinary practices carried out for routine clinical analysis, animal care or registration requirements. Since blood samples were not collected for experimental purposes, according to the Italian law (Decreto Legislativo 4/03/2014 n.26), the procedures do not require approval by an ethical committee. Written consent from the owners was not required because the identity of horses and owners cannot be established from the data presented in this work. DNA samples from endangered Equus species were shipped to Italy from the San Diego zoo together with the appropriate international CITES permit. Horse fibroblast cell lines were established from skin samples taken from animals not specifically sacrificed for this study; the animals were being processed as part of the normal work of the abattoirs.

### Preliminary *in silico* analysis of the polymorphism of the four ERE subfamilies

The consensus sequences of the ERE subfamilies ERE1 (accession number: D26566) [53], ERE2 [76], ERE3 [77], ERE4 [78] were downloaded from Repbase [27, 28] and used as queries for a BLAT search against the horse genome reference sequence (September 2007 Broad/equCab2.0 assembly) [51, 52]. For each ERE subfamily the 200 loci with the highest identity to their consensus sequence were identified. Their sequence was used as query for a BLAST search against the horse Trace database [54], which is a collection of short sequences (<1 Kb) generated during large-scale sequencing projects. From the Trace database we selected the dataset Equus caballus-WGS, which contains reads that were not included in the final assembly of the horse genome reference sequence. We then used the sequences flanking each ERE insertion as query to search for traces corresponding to the same loci but lacking the ERE insertion (empty alleles).

### Search of ERE1 loci characterized by insertion polymorphism in the horse genome reference sequence

Our preliminary search, based on the analysis of 200 loci from each ERE subfamily, showed that ERE1s have the highest proportion of empty alleles. We then focused further analyses on this subfamily.

In order to obtain a comprehensive catalog of ERE1 polymorphic loci in the horse genome reference sequence, we developed a pipeline using the C# programming language (Microsoft Visual Studio 2008) and Microsoft SQL Server 2008 as the database management system. The ERE1 consensus sequence downloaded from RepBase (accession number D26566) [53] was used as query for a BLAST search against the horse genome reference sequence (September 2007 Broad/equCab2.0 assembly) [79]. The BLAST search was performed using “megablast” as optimization algorithm and standard search parameters. Results were downloaded as hit table. Only the loci with identity to the consensus greater than 84 % were considered. To exclude loci that were subject to deletions or insertions, only the hits with length similar to that of the ERE1 consensus sequence (225 ± 10 bp) were considered. Since the coordinates of the hits inside the table were referred to contig sequences, they were converted into genomic coordinates using the conversion table “seq_contig.md” at [80]. ERE1s located inside unplaced regions were discarded. Since our method is based on similarity, ERE1s inserted inside other transposons could give rise to false positive hits because several uninterrupted transposons are scattered through the genome. Therefore, before starting the search for polymorphic loci we identified and discarded ERE1 elements inserted inside other transposons. To this purpose, we downloaded the list of the horse transposable elements from the site UCSC Genome Bioinformatics using the tool “Table Browser” [61, 62]. The list of transposons is found in the data table called “rmsk” (Group “Variation and Repeats”, Track “RepeatMasker”) that was generated using the software RepeatMasker [81] during the horse genome sequencing project [24]. The coordinates of each ERE1 were compared with those of the boundaries of other transposable elements. If an ERE1 interrupted a repetitive element the locus was discarded.

To identify empty alleles, for each locus we downloaded a 2.2 Kb sequence from UCSC Genome Browser [24, 82, 83] containing the transposon (about 225 bp), 1 Kb from the 5’ flanking region and 1 Kb from the 3’ flanking region. These sequences were then used as queries for a BLAST search [54] against the horse “Traces – WGS sequence” database. The BLAST search was performed using “megablast” as optimization algorithm and standard search parameters. If the hit contained a 225 ± 10 bp gap and was at least 98 % identical to the sequences flanking the transposon, it was considered an ERE1- locus. Only traces from the reference genome of Twilight were considered identifying them as belonging to “center_project number” G836. The specificity of each trace sequence was manually checked using BLAT [51, 52] and MultAlin [84, 85]. In order to focus on the loci inserted in single copy sequences, the ERE1 loci that were found at multiple positions during the BLAT search, and were probably located inside segmental duplications, were discarded. The complete list of single copy polymorphic ERE1 loci and the accession codes of the traces (Trace id) corresponding to the empty alleles is reported in Additional file 2: Table S2.

### *In silico* localization of ERE1 elements relative to genes

The position of ERE1 elements relative to horse genes was defined using the genomic coordinates of known horse validated and putative genes. Horse validated genes and their coordinates are listed in the data table “refGene” (assembly “Sep. 2007”) downloaded from the site UCSC Genome Bioinformatics using the tool “Table Browser” [61, 62]. The “refGene” table contains, among other information, the name of each gene, the coordinates of the transcription start and stop sites, the coordinates of the boundaries of each exon. Since the number of known horse genes is relatively small, we also included in the search the genomic coordinates of putative genes defined by sequence homology with those from human and bovine as listed in the data table “Other RefSeq (xenoRefGene)”. The data table (xenoRefGene) was downloaded from using the tool “Table Browser” [61, 62] and was used to define the coordinates of the beginning and end of putative genes in horse that are orthologous to those from human and bovine. This track was prepared by the UCSC genome browser group as described in the information page (https://genome.ucsc.edu/cgi-bin/hgTrackUi?hgsid=442242277_zw0eu9Hy93E8wlE62c8BxvE3BJox&c=chr11&g=xenoRefGene): as stated in the information page, this track shows known protein-coding and non-protein-coding genes for organisms other than horse. The RNAs were aligned against the horse genome using blat. This track was produced at UCSC from RNA sequence data generated by scientists worldwide and curated by the NCBI RefSeq project.

### Genomic DNA samples

Genomic DNA was extracted from blood or hair samples, or from cultured primary fibroblasts using standard protocols. The 30 *E. caballus* samples shown in Fig. 2 derive from: peripheral blood of 22 show jumping horses which, according to their pedigree chart, do not share common ancestors up to the third generation (they were also used for the analysis of the myostatin gene polymorphism shown in Table 1, see below); fibroblast cell lines established from the skin of 8 slaughtered animals which were shown to be unrelated by microsatellite analysis as described in [86]. The *E. asinus* samples derive from fibroblast cell lines established from the skin of 3 slaughtered animals. The *E. grevyi* sample derives from a fibroblast cell line purchased from Coriell Repositories and *E. burchellii* fibroblasts were a kind gift from Mariano Rocchi (University of Bari, Italy) [50, 87]. *E. zebra hartmannae*, *E. kiang* and *E. hemionus onager* fibroblasts were provided by Oliver Ryder (Genetics Division of San Diego Zoo, San Diego, California, USA) [48]. DNA samples from Quarter Horses, Andalusian, Norwegian Fjord, Icelandic Ponies (Table 1) and *E. przewalskii* (Fig. 2 and Table 1) were provided by Cecilia Penedo (UC Davis, California, USA). Lipizzaner DNA samples (Table 1) were described in [88]. The 30 Show Jumpers in Table 1, which comprise the 22 *E. caballus* individuals of Fig. 2, were animals kept in Italian sport riding stables and competing at the National and International level; they derived from different stud farms in Italy, France, Germany, Holland, Belgium and were chosen by the owners for their show jumping aptitude. Genomic DNA from Italian Trotters and Italian Thoroughbreds was extracted from blood spotted on FTA® filter papers (Whatman Bioscience, Cambridge, UK). All samples came from horses belonging to the Italian Stud Book of MiPAAF (Ministero Delle Politiche Agricole Alimentari e Forestali). The performance information were provided by ANAC (Associazione Nazionale Allevatori Cavalli Purosangue).

### PCR and SNP analysis

Eighty ERE1 insertions with different degrees of identity relative to the consensus sequence were randomly selected from the list of 27,396 loci obtained by *in silico* analysis. The coordinates of the 80 loci are reported in Additional file 2: Table S4A together with the sequence of the primers deduced from the sequences flanking the transposon (Additional file 2: Table S4A). Twenty ng of genomic DNA were used as template for PCR experiments performed in a 10 μl-final volume with 8 pmoles of each primer, 0.2 mM dNTPs, 1× Green Buffer (Promega) and 0.4 units of GoTaq DNA polymerase (Promega). After a denaturation step at 95 °C for 2 min, the following amplification cycle was performed 3 times: 95 °C for 50 s, appropriate annealing temperature (Additional file 2: Table S4A) for 45 s, 72 °C for 1 min. The first 3 cycles were followed by 27 cycles: 95 ° C for 30 s, appropriate annealing temperature for 35 s, 72 °C for 1 min. Final extension was carried out at 72 °C for 5 min. PCR products were checked by electrophoresis in 1 % agarose gel.

To analyze the ERE1 insertion polymorphism at the myostatin promoter, we amplified genomic DNAs using primers from the sequences flanking the insertion site (MyostProm-F0 and MyostProm-R, Additional file 2: Table S4B). The expected length of the PCR products from the ERE1+ and the ERE1- alleles were 441 and 214 bp, respectively. The reactions were carried out as described above.

The Analysis of SNP g.66493737C > T was performed using the “Custom TaqMan SNP Assay” (Applied Biosystems) on a 7500 Fast Real Time PCR Instrument.

### Preparation of plasmids for reporter gene assay

In order to clone the entire promoter and the transcription start site of the myostatin gene we PCR-amplified the locus chr18:66495283–66497324 (equCab2.0) from the genomic DNA of a horse heterozygous for the ERE1 insertion.

PCR reaction was performed using the primers MyostProm-F and MyostProm-R (Additional file 2: Table S4B), which contain *Hind*III and *Bam*HI restriction sites, respectively. After a denaturation step at 95 °C for 2 min, the following amplification cycle was repeated for 30 times: 94 °C for 40 s, 65 °C for 40 s and 72 °C for 4 min. The final extension was carried out at 72 °C for 10 min. The reaction products corresponding to the ERE1- and the ERE1+ allele (2058 and 2285 bp, respectively) were separated by electrophoresis on 1 % agarose gel and purified using the Wizard SV Gel and PCR Clean-Up System (Promega). The two alleles differed only for the presence of the ERE1 element and the target site duplication (see Fig. 3).

The purified PCR products were digested with *Hind*III and *Bam*HI and then cloned, upstream of the enhanced Green Fluorescent Protein (*eGFP*) cDNA, into an expression vector that was previously constructed in our laboratory [89]. Our vector contains the puromycin and ampicillin resistance genes. All constructs were checked by Sanger sequencing.

### Cell culture and transfection

Horse Immortal Fibroblasts [75] and HeLa (human cervical carcinoma) cells were cultured in high-glucose D-MEM supplemented with 10 % fetal calf serum (Euroclone), 2 % non-essential amino acids, 2 mM L-glutamine and 1× penicillin-streptomycin (Sigma). For primary fibroblast cell lines, the culture medium was supplemented with 20 % fetal calf serum. Cells were routinely cultured at 37 °C in 5 % CO_2_.

Plasmid DNA for promoter reporter assays was prepared using QIAGEN Plasmid Midi kit. Transfections were carried out using the Lipofectamine 2000 reagent (Invitrogen) according to the manufacturer’s protocol.

Twenty-four hours post-transfection, cells were selected adding 300 ng/ml (horse immortal fibroblasts) or 1 μγ/ml (HeLa cells) puromycin to the medium. Cells were cultured with selective medium until the emergence of drug-resistant colonies, that is 3 weeks for horse fibroblasts and 2 weeks for HeLa cells. Pools of about 50 colonies were obtained and grown as stably transfected cell populations.

### Western Blot experiments

Protein extracts were prepared from samples three million cells as follows: the cells were washed twice with ice cold 1xPBS, resuspended in lysis buffer (50 mM Tris–HCl pH 6.8, 86 mM β-mercaptoethanol, 2 % SDS) and boiled for 10 min. Proteins were separated by 10 % SDS-PAGE and transferred to nitrocellulose membranes (Amersham Protran Premium 0.45 μm NC) through wet transfer. Membranes were incubated with a rat monoclonal antibody against eGFP (Chromotek, code 3H9), diluted 1:1000, and with a mouse monoclonal antibody against tubulin (NeoMarkers, Ab-4, code MS-719-P1ABX), diluted 1:3000. Secondary antibodies, conjugated to horseradish peroxidase, were a chicken anti-rat IgG-HRP (Santa Cruz Biotechnology, code sc-2956), diluted 1:5000, and an ImmunoPure goat anti-mouse monoclonal (H + L) (Pierce, code 31430), diluted 1:10,000. Detection was performed using Immun-Star WesternC Kit (Bio-Rad) according to the manufacturer’s protocol. Pre-incubation of the membranes and dilutions of the antibodies were performed in 1xPBS containing 0,05 % Tween20 and 7.5 % skim milk.

### eGFP fluorescence analysis

Cells for eGFP fluorescence analysis were grown on coverslips (24 × 24 mm), washed with cold 1xPBS and fixed in 2 % paraformaldehyde in PBS for 10 min. Fixed cells were then stained with DAPI (4,6-diamidino-2-phenylindole) and observed with a ZEISS Axioplan fluorescence microscope at 63× magnification. Pictures were captured using a CoolSNAP CCD camera (RS Photometrics) and processed using the software IPLab 3.5.5 (Scanalytics inc).

### RNA preparation and quantitative RT-PCR

Total RNA from transfected HeLa and horse fibroblast cells was extracted using TRizol Reagent (Invitrogen) according to the manufacturer’s protocol. The extracted RNA was purified using the RNA Clean & Concentrator-25 kit (Zymo Research) and treated three times with RQ1 RNase-free DNase (Promega).

For quantitative RT-PCR experiments we reverse transcribed 2.5 μg of total RNA using oligo-d(T)_17_ primers and Revert Aid Premium First Strand cDNA synthesis kit (Fermentas) according to the manufacturer’s protocol.

The cDNA was PCR amplified using GoTaq qPCR Master Mix (Promega) containing the appropriate oligonucleotides (Additional file 2: Table S4B). Oligonucleotides eGFP-F and eGFP-R were used to detect the eGFP transcript. *GAPDH* (glyceraldehyde 3-phosphate dehydrogenase, primer pair GAPDH-F and GAPDH-R) or *PRKCI* (protein kinase C iota, primer pair humcavPRKC-RealT-F and cavPRKC-RealT-R) were used as control genes for quantitative RT-PCRs carried out with the cDNA from HeLa cells or horse immortal fibroblasts, respectively. Each sample was prepared in triplicate. Negative controls (No template controls, NTCs) were included in the experiments. Reactions were carried out using an Opticon 2 System instrument (MJ Research). Cycling parameters comprised an initial denaturation at 95 °C for 2 min followed by 50 cycles at 95 °C for 15 s, 62 °C for 30 s and 72 °C for 30 s coupled to fluorescence detection. Experiments were repeated twice for each transfected cell line. Data were analyzed with the Opticon Monitor 3 software. Levels of expression were calculated using the standard ΔΔCq method [90], the level of expression in cells transfected with the plasmid containing the wild type allele was used as reference.

### Statistical analysis

The correlation between the percentage of identity of the ERE1 loci and the natural logarithm of the frequency of polymorphic loci in each class was tested calculating Pearson’s product moment correlation coefficient.

The significance of the difference of the allelic frequencies at the myostatin promoter in the populations of Elite and Unselected Thoroughbreds was tested using a Chi-Square test goodness of fit. The allelic frequencies in the 75 Unselected Thoroughbreds were adopted as expected values.

The significance of the correlation between the Best Race Distance and the genotype of the 117 Elite Thoroughbreds for the ERE1 insertion at the myostatin promoter was tested using a Chi-Square test for independence.

All statistical analyses were performed using R [91].

### Availability of supporting data

The data sets supporting the results of this article are included within the article and its additional files.

## Additional files

Additional file 1:
**Table S1.A** lists the 45,713 loci identified using the ERE1 consensus sequence deposited at the RepBase database as query for a BLAST search against the horse genome reference sequence. **Table S1B** reports the 34,131 ERE1 loci with sizes similar to the ERE1 consensus (225 ± 10 bp) and with minimum identity to the consensus of 84 %. **Table S1.C** lists the 27,396 ERE1 loci that are not located inside other repetitive elements. (XLSX 3293 kb) Additional file 2:
**Table S2.** lists the ERE1 polymorphic loci identified in the horse reference genome sequence. **Table S3.** reports the frequency of ERE1 polymorphic loci in eight classes of ERE1 elements grouped according to consensus identity. The values reported in this table were used to draw Fig. 1. **Table S4A** lists the genomic position of the 80 ERE1 loci analysed in Fig. 2 and the sequence of the primers used for each locus. Table S4B lists the primers used to clone the myostatin promoter region and those used to perform quantitative RT-PCR experiments for reporter gene assay. (PDF 184 kb)

## Abbreviations

BLAST: Basic Local Alignment Search Tool
BLAT: BLAST-Like Alignment Tool
DAPI: 4’,6-diamidino-2-phenylindole
EAS: Equus asinus
EBU: Equus burchellii
eGFP: Enhanced Green Fluorescent Protein
EGR: Equus grevyi
EZH: Equus zebra hartmannae
EHO: Equus hemionus onager
EKI: Equus kiang
ERE: Equine Repetitive Element
GAPDH: Glyceraldehyde 3-phosphate dehydrogenase
LINE: Long INterspersed Element
Mya: Million years ago
NTC: No Template Control
NUMT: NUclear sequences of MiTochondrial origin
PCR: Polymerase Chain Reaction
PRKCI: Protein kinase C iota
RT-PCR: Real Time Polymerase Chain Reaction
SINE: Short INterspersed Element
SNP: Single Nucleotide Polymorphism.
