# Supplementary material for: Genome-wide evolutionary and functional analysis of the Equine Repetitive Element 1: an insertion in the myostatin promoter affects gene expression
Source: BMC Genet. 2015 Oct 26;16:126. doi: 10.1186/s12863-015-0281-1 (PMC4623272; doi:10.1186/s12863-015-0281-1)
Supplement: Additional file 2: — Table S2. lists the ERE1 polymorphic loci identified in the horse reference genome sequence. Table S3. reports the frequency of ERE1 polymorphic loci in eight classes of ERE1 elements grouped according to consensus identity. The values reported in this table were used to draw Fig. 1. Table S4A lists the genomic position of the 80 ERE1 loci analysed in Fig. 2 and the sequence of the primers used for each locus. Table S4B lists the primers used to clone the myostatin promoter region and those used to perform quantitative RT-PCR experiments for reporter gene assay. (PDF 184 kb) [file 12863_2015_281_MOESM2_ESM.pdf]

Table S2: ERE1 polymorphic loci identified in the horse reference genome sequence

Chromosome Number	Starting nt of ERE1 element	Length (bp)	Identity to ERE1 consensus (%)	Empty locus Trace Id
1	119005334	224	96-98	ti1215431275
1	122955009	225	96-98	ti1338462463
1	132798463	224	96-98	ti1242704828
1	139244728	226	98-100	ti1345450582
1	142524414	222	96-98	ti1441728429
1	157968703	224	96-98	ti1240572182
1	159183823	225	96-98	ti1215478173
1	160438645	225	94-96	ti1437204650
1	162286651	224	98-100	ti1396137832
1	166968129	224	98-100	ti1219925157
1	168410626	224	96-98	ti1314566604
1	175481345	224	96-98	ti1328444586
1	175975035	224	94-96	ti1338461979
1	21353720	226	96-98	ti1237582549
1	27588859	224	94-96	ti1313009302
1	32232170	218	98-100	ti1291079063
1	33436660	224	96-98	ti1420425925
1	39976917	221	94-96	ti1396085618
1	61887990	224	98-100	ti1202342996
1	67072133	223	94-96	ti1326197361
1	70334150	224	96-98	ti1398714699
1	77780664	225	96-98	ti1204232417
1	95896927	224	96-98	ti1238111707
1	96482133	224	96-98	ti1287615055
1	99651451	223	86-88	ti1273982445
2	117955177	228	94-96	ti1245232418
2	118880340	227	96-98	ti1398775766
2	15674823	224	98-100	ti1442639653
2	16837108	224	98-100	ti1290032208
2	24635971	224	98-100	ti1285029298
2	27587258	224	94-96	ti1290665863
2	40543240	224	92-94	ti1275531176
2	44280304	225	96-98	ti1703048483
2	56931292	225	98-100	ti1271517840
2	57190462	223	94-96	ti1430470010
2	59234471	223	88-90	ti1399965987
2	60610997	225	96-98	ti1210325185
2	62508910	224	96-98	ti1226005223
2	67762614	224	98-100	ti1267874663
2	73001824	224	96-98	ti1304862300
2	74331951	221	96-98	ti1269042459
2	82007973	228	96-98	ti1239408604
2	84843183	226	96-98	ti1249998358
2	92382557	224	96-98	ti1232736755
3	2176049	224	98-100	ti1204044317
3	241512	225	96-98	ti1239179539
3	3900246	227	92-94	ti1338465145
3	52180011	224	94-96	ti1237984079
3	62376779	223	94-96	ti1273589568
3	73013621	225	94-96	ti1221903818
3	80119010	227	90-92	ti1224686080
3	81186227	225	96-98	ti1200696546
3	90166388	225	96-98	ti1326246127
3	90642742	224	98-100	ti1413253074
3	91008739	225	98-100	ti1202855803
3	91970934	224	96-98	ti1288169546
3	98431604	224	98-100	ti1419336465
4	103765160	227	94-96	ti1198700043
4	10450249	224	96-98	ti1215562135
4	107479407	224	96-98	ti1309136705
4	12481160	224	96-98	ti1438236705
4	13156659	224	98-100	ti1218570252
4	23439591	226	96-98	ti1234739903

4	27683720	224	98-100	ti1280064423
4	28712512	225	94-96	ti1435937300
4	3434498	223	96-98	ti1329174193
4	3918513	223	86-88	ti1296885026
4	42877092	223	98-100	ti1304004617
4	44838238	225	96-98	ti1202379653
4	51315150	225	98-100	ti1355698221
4	8749510	225	96-98	ti1435965891
4	97747106	224	96-98	ti1267330187
4	99923581	226	96-98	ti1213947348
5	31087230	225	94-96	ti1268865810
5	3470758	223	98-100	ti1400624301
5	58715377	228	94-96	ti1299296993
5	59360885	224	96-98	ti1278981694
5	74743823	224	96-98	ti1326935948
5	75177823	224	94-96	ti1333640978
5	79823463	225	96-98	ti1221085110
5	80498138	224	96-98	ti1243155745
5	80597941	223	96-98	ti1305775971
5	80782761	235	92-94	ti1202439902
5	80914308	224	96-98	ti1378485385
5	88501073	229	94-96	ti1248655775
5	89712438	229	96-98	ti1237340948
5	94114664	224	96-98	ti1200807395
5	97263422	224	96-98	ti1270323771
6	10663755	223	96-98	ti1265189583
6	12176270	220	92-94	ti1301064970
6	12763260	225	96-98	ti1240123909
6	13819959	221	94-96	ti1227493921
6	21199080	224	96-98	ti1399132648
6	41537845	225	98-100	ti1249463860
6	44444414	223	96-98	ti1236305456
6	45760316	225	94-96	ti1286226414
6	48485829	223	96-98	ti1248383123
6	51263502	226	96-98	ti1215574974
6	52813781	221	98-100	ti1229323066
6	53656327	226	94-96	ti1417057896
6	5653511	226	92-94	ti1328402110
6	58980132	221	92-94	ti1240096121
6	59868416	224	94-96	ti1284882493
6	61460413	224	98-100	ti1202332125
6	66975553	225	92-94	ti1266006189
6	72303843	224	96-98	ti1279361876
6	73939623	227	96-98	ti1285098148
6	76728714	224	98-100	ti1286851041
7	11307152	224	96-98	ti1332201425
7	11844272	225	92-94	ti1221021233
7	14179208	225	96-98	ti1266640853
7	16912832	224	98-100	ti1245088446
7	23126555	225	94-96	ti1440890833
7	25505150	224	98-100	ti1253045803
7	26092808	227	96-98	ti1286856038
7	27731067	224	96-98	ti1278678482
7	29321347	223	98-100	ti1394962626
7	31669112	223	98-100	ti1250294726
7	33473476	224	96-98	ti1248498818
7	38489007	224	96-98	ti1292480481
7	48261012	224	96-98	ti1238984660
7	54155447	225	96-98	ti1209933147
7	5436822	224	98-100	ti1285107480
7	55141290	225	94-96	ti1425098581
7	56620838	223	98-100	ti1255958612
7	57857701	224	96-98	ti1265345686
7	58347059	224	96-98	ti1222841744
7	82674355	223	96-98	ti1238938996
7	8565596	227	94-96	ti1221812930

7	89678654	224	98-100	ti1262627678
8	11633866	225	96-98	ti1289841497
8	12100052	224	98-100	ti1234823510
8	18089584	222	96-98	ti1250008305
8	24968742	223	98-100	ti1358334196
8	30701414	224	96-98	ti1288513453
8	31516043	221	94-96	ti1258109360
8	37519891	224	98-100	ti1310637159
8	42507193	224	96-98	ti1258294295
8	56166838	224	94-96	ti1287890059
8	59861493	225	98-100	ti1210067439
8	64243778	224	96-98	ti1266570036
8	69992406	224	98-100	ti1426344283
8	931788	226	94-96	ti1411528637
8	938844	224	98-100	ti1204162804
9	1711234	225	94-96	ti1406382249
9	18864821	224	96-98	ti1305768547
9	2461151	225	98-100	ti1278652384
9	3098990	224	94-96	ti1210146717
9	33461006	224	96-98	ti1199270370
9	33580270	224	94-96	ti1269929100
9	39719615	224	96-98	ti1232224387
9	4072161	218	96-98	ti1220169530
9	4883744	226	96-98	ti1286145579
9	50244663	224	94-96	ti1335723842
9	51692753	225	96-98	ti1245125288
9	55324528	224	98-100	ti1237609843
9	56134210	224	98-100	ti1222901243
9	62995591	226	98-100	ti1358256358
9	9520837	224	96-98	ti1248929480
10	13059866	224	96-98	ti1307623530
10	18394842	215	98-100	ti1305306395
10	20518239	224	98-100	ti1290880713
10	21961492	224	96-98	ti1424556107
10	22258740	224	96-98	ti1258019208
10	22494796	216	94-96	ti1205578676
10	22781060	224	98-100	ti1417782691
10	25884255	223	94-96	ti1305892573
10	31738682	224	96-98	ti1300931745
10	32453942	224	96-98	ti1326172691
10	34153429	228	96-98	ti1355714551
10	36792607	224	94-96	ti1406858493
10	41196338	224	94-96	ti1332088770
10	41590489	224	96-98	ti1224810489
10	4965178	224	96-98	ti1334263517
10	51884399	220	94-96	ti1349503816
10	52690163	224	98-100	ti1288124161
10	53127486	222	96-98	ti1428118731
10	54587849	224	98-100	ti1268332618
10	55566843	225	96-98	ti1298436769
10	57680530	224	98-100	ti1332216534
11	13417958	223	96-98	ti1237947898
11	20006805	224	98-100	ti1437476205
11	22906347	224	96-98	ti1325616779
11	27008920	224	98-100	ti1242886637
11	43062908	229	96-98	ti1229305455
11	50731773	224	94-96	ti1442315286
11	52900275	227	96-98	ti1256415810
11	55396842	224	96-98	ti1200730164
11	9466680	215	98-100	ti1425098093
12	12227765	224	98-100	ti1345902327
12	13172534	227	96-98	ti1256238725
12	31371137	226	96-98	ti1297703596
12	6222671	224	96-98	ti1436875729
13	17161483	225	96-98	ti1304555892
13	22814220	226	96-98	ti1345896853

13	23855769	224	98-100	ti1261298353
13	24455422	225	98-100	ti1306101210
14	11298697	225	96-98	ti1221813933
14	16660998	224	88-90	ti1266695635
14	189088	225	92-94	ti1242875531
14	29626379	216	94-96	ti1322392930
14	511793	226	92-94	ti1209052330
14	514896	224	96-98	ti1207497256
14	6840876	224	98-100	ti1202860165
14	70182536	225	96-98	ti1287904556
14	71027041	227	96-98	ti1236767556
14	71940008	225	96-98	ti1438243933
14	73221748	224	98-100	ti1303971451
14	73589300	227	96-98	ti1204501927
14	74188792	224	96-98	ti1435526371
14	78348337	225	96-98	ti1254221096
14	79384039	225	96-98	ti1258009121
14	79782840	223	98-100	ti1349130049
14	80605833	225	96-98	ti1440908861
14	81940943	225	96-98	ti1215485810
14	8358658	224	94-96	ti1328915514
14	85258435	225	96-98	ti1326217603
14	85639757	223	96-98	ti1322726913
14	87667277	225	96-98	ti1239336724
14	89134944	224	98-100	ti1436937731
15	14419374	224	98-100	ti1421182121
15	25832992	229	94-96	ti1260090013
15	26506464	224	98-100	ti1425099288
15	26720183	218	92-94	ti1220009209
15	27784339	224	98-100	ti1210615362
15	37716242	224	96-98	ti1245284145
15	42711057	226	94-96	ti1299383147
15	42891559	225	96-98	ti1316828752
15	43278199	225	98-100	ti1313797400
15	50244574	225	96-98	ti1232227665
15	5129237	223	96-98	ti1399171638
15	5513680	228	96-98	ti1258093947
15	65169650	223	94-96	ti1421469726
15	65574226	227	90-92	ti1217440123
15	6776371	224	96-98	ti1249441937
15	68225447	221	84-86	ti1264620844
15	74802667	223	92-94	ti1233578863
16	20756470	223	96-98	ti1289942952
16	24970114	224	98-100	ti1252793138
16	27256368	224	96-98	ti1279308349
16	28499191	226	94-96	ti1219608947
16	31043681	220	96-98	ti1202337731
16	39135794	224	96-98	ti1402556075
16	44437633	224	96-98	ti1405279572
16	47696933	225	96-98	ti1263773537
16	61908506	224	98-100	ti1431806600
16	63846022	221	96-98	ti1332279692
16	72898188	224	98-100	ti1236148214
16	74617640	225	94-96	ti1237340942
16	78499590	227	96-98	ti1327066478
17	60545620	226	96-98	ti1347806805
18	12983530	224	98-100	ti1417192457
18	13348	224	96-98	ti1326184109
18	37445	224	96-98	ti1441738502
18	55984160	226	96-98	ti1202332936
18	57184812	224	98-100	ti1231436995
18	7008257	224	98-100	ti1220017554
18	75488066	231	92-94	ti1278788560
18	76252565	223	94-96	ti1206899165
18	8514401	224	98-100	ti1303988461
19	1932713	225	96-98	ti1252966405

19	2273544	225	96-98	ti1289551414
19	22792573	221	94-96	ti1278695658
19	27297728	224	96-98	ti1262053162
19	32088581	233	94-96	ti1200859656
19	33226042	224	98-100	ti1446174989
19	39537161	224	96-98	ti1278792001
19	48975824	226	96-98	ti1306169646
19	4927606	229	92-94	ti1250704724
19	4969085	222	96-98	ti1299026374
19	5075319	225	96-98	ti1245154484
19	57812538	229	96-98	ti1299014824
20	12785940	223	94-96	ti1290457620
20	14729391	224	98-100	ti1398515213
20	19787980	223	94-96	ti1263142519
20	58632555	226	94-96	ti1206004494
20	59249264	230	96-98	ti1212467201
20	64126751	227	94-96	ti1273019382
21	10586969	225	96-98	ti1200691774
21	19996843	224	92-94	ti1222752835
21	20178849	224	96-98	ti1237029722
21	22010690	225	92-94	ti1236308792
21	24166342	225	96-98	ti1435990485
21	25482692	220	94-96	ti1236141254
21	25794012	224	96-98	ti1248371962
21	35863486	225	94-96	ti1256096842
21	38325726	224	94-96	ti1210807462
21	38601590	225	94-96	ti1221823280
21	40359821	224	98-100	ti1426249001
21	43215421	224	98-100	ti1222571614
21	8476093	224	96-98	ti1263462953
21	8874069	225	96-98	ti1251096377
22	18454140	224	94-96	ti1397518957
22	23999964	223	96-98	ti1310305737
22	26642475	224	98-100	ti1221550112
22	33852963	226	96-98	ti1384043603
22	35806798	225	98-100	ti1250892478
22	37279717	219	94-96	ti1398773880
22	38865913	224	96-98	ti1221038723
22	39104698	224	94-96	ti1267886747
22	39114540	235	90-92	ti1213969742
22	41960683	225	96-98	ti1257262422
22	42245385	224	98-100	ti1209052772
23	10368196	225	96-98	ti1386809449
23	17693567	224	98-100	ti1306317813
23	286305	225	96-98	ti1227274516
24	12346382	226	98-100	ti1287850350
24	12906139	217	96-98	ti1248692064
24	21124791	221	94-96	ti1406331557
24	28519561	224	98-100	ti1436564687
24	30445697	223	96-98	ti1278735757
24	30983772	227	94-96	ti1202496348
24	38009047	224	96-98	ti1279738654
25	13457288	225	94-96	ti1333614555
25	19709759	224	98-100	ti1249447118
25	21008469	224	96-98	ti1283532523
25	26940190	226	98-100	ti1438222131
25	3064231	224	98-100	ti1285043324
25	30645818	223	98-100	ti1702766444
25	30753712	226	96-98	ti1261923421
25	4624489	219	94-96	ti1279249740
25	8046189	225	98-100	ti1257217673
26	11218230	224	96-98	ti1279521961
26	17069878	224	98-100	ti1220492373
26	19129874	223	96-98	ti1198689771
26	37236715	218	92-94	ti1424295136
26	37867327	223	96-98	ti1227789425

26	38282741	226	96-98	ti1227642740
26	40960045	224	98-100	ti1307041522
26	7118978	224	94-96	ti1292099277
26	9018676	224	96-98	ti1437769026
27	11195786	225	96-98	ti1242372808
27	21083583	224	96-98	ti1230640407
27	27143364	224	98-100	ti1221521548
27	313031	224	96-98	ti1237367904
27	33493115	224	94-96	ti1219228592
27	34247629	223	98-100	ti1290883375
27	35040169	224	96-98	ti1269929449
28	16949023	224	94-96	ti1219164392
28	189945	224	98-100	ti1328376684
28	19262329	226	96-98	ti1268405120
28	27454993	223	96-98	ti1329521775
28	3074054	224	94-96	ti1237030428
28	41109467	224	92-94	ti1227328929
28	8159921	225	96-98	ti1227150032
29	1084589	227	96-98	ti1243167709
29	1095880	226	96-98	ti1423825932
29	13308120	223	94-96	ti1279735269
29	24276432	224	94-96	ti1257246899
29	26188569	222	96-98	ti1426334217
29	4006851	224	98-100	ti1349079930
30	10488424	224	96-98	ti1232091203
30	17932193	224	96-98	ti1406982234
30	19245146	224	96-98	ti1271116250
31	11856502	224	92-94	ti1209906429
31	6881049	225	94-96	ti1345647408
31	7599351	224	96-98	ti1212469932
31	7829288	225	92-94	ti1253863294
31	7837016	225	96-98	ti1240059411
X	110970420	224	96-98	ti1266539395
X	111952812	224	96-98	ti1248963206
X	123009868	224	96-98	ti1273695957
X	15978730	224	98-100	ti1286769416
X	18252122	226	96-98	ti1219988022
X	19214998	225	98-100	ti1305370866
X	19385329	226	96-98	ti1279737282
X	27381528	223	94-96	ti1426247430
X	30306902	224	94-96	ti1416583632
X	34537236	220	96-98	ti1309709575
X	57680224	223	98-100	ti1201016218
X	57712690	224	92-94	ti1229339488
X	68585085	224	98-100	ti1427928591
X	8188991	222	98-100	ti1328078818
X	83890064	220	92-94	ti1267888700
X	86048557	229	94-96	ti1202695832
X	86936672	223	98-100	ti1202321008
X	98249065	223	98-100	ti1263471996

Table S3: frequency of ERE1 polymorphic loci in 8 classes of elements grouped according to the % of identity to the consensus

Identity to ERE1 consensus (%)	Number of polymorphic loci in Twilight	Total number of loci	% polymorphic loci
84-86	1	988	0,10
86-88	2	1264	0,16
88-90	2	1217	0,16
90-92	3	2580	0,12
92-94	22	6180	0,36
94-96	72	7246	0,99
96-98	180	5866	3,07
98-100	95	2055	4,62
	377	27396	

Table S4A: primers used for the PCR experiments

Locus	Genomic position of the amplified sequence	Identity to ERE1 consensus (%)	Forward primer (5' -> 3')	Reverse Primer (5' -> 3')	Ta (°C)	Product length (bp) (ERE1+ allele)	Product length (bp) (ERE1- allele)
1	chr1:13834310-13834801	98-100	CTCCTAGGCAGGTCACACCA	CATCTTCATGCCCAGTGACA	61	492	267
2	chr2:62092638-62093074	98-100	TCTCGGATCTCAACCACTTC	TGTGCTTGGTCTTTAGTCCCT	61	437	212
3	chr3:93335162-93335732	98-100	GATGACCCAACTAAAATGGTG	AGCTGGATAATGCTGTCCTT	61	571	346
4	chr5:43214771-43215433	98-100	TCATGCAGGTGGAGACGTTC	CTTAGGAGACAGGCCATTTGCT	60	663	438
5	chr5:48342542-48342970	98-100	TGCTGGACTTACGAAGGAAGA	CCTTCAAATGGGTAGTAGACTTCTC	61	429	204
6	chr6:51284262-51285093	98-100	TTACTTAGGGTGAGGATGCTG	TGACACATACTTTTGCTGACAC	61	832	607
7	chr6:78964068-78964653	98-100	CCACAACTTGCTGGTGAGAT	CTGAGTGTCCTAATTCTGTTTTGC	61	586	361
8	chr7:33172951-33173470	98-100	AACTCAGCAGGGATCTAGGTGT	GCTGAGTACCTGTGGTGACTTC	61	520	295
9	chr8:12099909-12100673	98-100	CTCCTCTTTGCTGTCCTGTG	CCCTTATCCTCTGCTATATCCCT	61	765	540
10	chr8:39891927-39892409	98-100	TTTCATCTTAGAGGCCAGTGCT	CTTTGTGCCCTTTGCCTCTC	61	483	258
11	chr10:80047366-80048169	98-100	TGTTTCCCACCACTCTGCTAG	CATGTCAGGGAATCTTTGGACTC	61	804	579
12	chr12:4615691-4616659	98-100	ATACAACAGCACGTGTTCCAAG	CCTGCCAAAGTTAAACTACCTCA	63	969	742
13	chr16:11773281-11773741	98-100	GCAGGAATCAGTGCAAAGTG	CTTTTATGTTTTCGTCATTGATGG	61	461	236
14	chr16:84679431-84680112	98-100	AACGGAAGTGTGCGAAGAAG	GGGATTGGCTTTCCACAAGT	61	682	457
15	chr17:74637428-74638192	98-100	GTCAATTCTTGTGTTGGTAGAGG	GTCGCTCTTTTAAATGTCCTG	61	765	540
16	chr19:25857418-25857871	98-100	GTTACAGGACTAGGAGCCAGAT	AAAGGATGATGCAAATGTATGAT	60	454	229
17	chr23:50238273-50239182	98-100	CTTTCGTCTTGCATGAGTACTCTG	ATACACCTGAAACTCCCCATCA	61	910	685
18	chr24:31125203-31125724	98-100	ACCTAATCCATACGGTCATCG	AGGTTGGTCATGTCTTCCATC	61	522	297
19	chrX:89385279-89385855	98-100	GCCAAGTTTAAATGCGCTTTAG	GGTTTTGACTGAAGGGAGAAGAG	60	577	352
20	chrX:93395396-93395996	98-100	GGAAGGTAGGGGAAATGCTTG	AAGACAATCTGGTTACGTGAGGA	61	601	376
21	chr1:40320790-40321431	95	CTTCAGCACTGGTTGCATCC	AATATTGGTGTGCCTCTCTTGG	61	642	417
22	chr1:67622766-67623417	95	ACGAAATGAGCAGAGAAGTGG	GGAACTGTGGACAAGTGCCTA	63	652	427
23	chr1:83457556-83458192	95	CCACAGCATGGAGTTATTGCC	GGAGGAAGGGTGGAAGGAGT	61	637	412
24	chr1:124526475-124526988	95	CCCAAGACCAGCTCATTGAT	CAGGAGAGAGCGTGACTGTG	63	514	289
25	chr1:168265568-168266090	95	CCCTGGTATCGATTTTCATCA	CTCTTCATCAGGGTAGCACG	63	523	298
26	chr8:91198232-91198736	95	CAAGGGAAGACTCTGATGGTG	CCAAGCAGTGATGTACAATCGT	63	505	280
27	chr9:32501988-32502633	95	CGGTAAAAATGAAAAATGCTGAGA	AATAACTGTTTGATCTGGATTGGAC	60	646	421
28	chr10:57553847-57554425	95	CCACCCACCCATTCTCCTAC	CTACGTGGGCGAGAACTCACT	61	579	354
29	chr11:16575664-16576231	95	GCTTTATTTCCCATGCCAA	CAACCCCATGTCAGATTTCC	60	568	343

30	chr14:33630411-33630959	95	AGTGTTGGTCATGTGGGTG	TCTTTCTTCTGTGGGGAGGA	60	549	324
31	chr16:8268290-8268925	95	GCCCTCCCACATTTTATGACT	GGATCATTTCCGCCAACTTC	61	636	411
32	chr16:16273689-16274224	95	CTATGGTGATGACAGGGAGAAAG	TTGAAGTCGCCTGTAAATAAGAGT	59	536	311
33	chr17:54006602-54007104	95	CATGCTACCTCTCATCCAGTCTC	CTTAATTCAATGTTCTGCGCTGTT	61	503	278
34	chr19:14762154-14762846	95	TCAGGGTCTGCTTTTTCTGGT	TTGTTTTGCCTCCAAACTCC	60	693	468
35	chr20:37147086-37147654	95	ATGGGACCTTAGACGGTTGA	GCTGCTGACTGTCCGAGAA	63	569	344
36	chr21:6856935-6857652	95	CCCTTGACTTCTCTCTCCTCTG	GCTTTTGGAAGAAGGCACC	63	718	493
37	chr23:39318706-39319453	95	TGTATGTCCTCAGGCATGGAA	TCCCAGGAGCAATGATGAAG	63	748	523
38	chr24:10750024-10750648	95	ATGGAAGGGATGTGACAAAGGT	TCCTCAACAACATTGGAATGC	59	625	400
39	chr31:18215793-18216378	95	CAACATGGTCATCTCCCAAAG	GGCGGGTTTCTAAGCACTAA	63	586	361
40	chrX:56172715-56173308	95	TGATTGCCCTAAAAGTATGCATTC	ACTTTCGGAGGAGGTGGTGA	61	594	369
41	chr1:152751141-152751698	90	TGGAGAAATGAGGTGATGGT	TCATGTGTGAGGAGTGCAGT	61	558	333
42	chr4:18841617-18842593	90	CGGTTGGTTGAGTCAGTGGA	AGAGGCATGTGCTAGCTGGT	61	977	752
43	chr5:1086474-1087298	90	TAGCGGCTTGACCTTTGTCCTTC	CCAAGAGACTCCTACACCTCATCC	64	825	552
44	chr5:32433857-32434264	90	CGTTGAAGGAAGCTCTGTCT	AAAGCTGGCAGTGATGGTGT	61	408	183
45	chr6:67851272-67851673	90	CCATCTCCTCAGCCTTATGAA	GTTGCCGGGATTGTACATTG	61	402	177
46	chr7:82313072-82313611	90	CCTTTTCAGATGCACAAGTGATA	AACCCACAGAAGCTACCCT	60	540	315
47	chr9:45909904-45910769	90	GAATCTTGGTGCCCTGGTGA	ACTGTGGCCTTGACATTGTGC	64	866	641
48	chr9:75532067-75533153	90	CTTGAAGACTGGTGCCTCCTGA	GTGGAGTGGCAGTGGAGATAGAG	68	1087	839
49	chr10:28296962-28297609	90	GGTGTGGGATACCAGCAGAA	TGGCTGGATTAGATATGGAAGG	61	648	423
50	chr11:25229211-25229707	90	GCTACACTGTAGGACCTGGATGA	GACAGACCTTCCAAGATGATG	61	497	272
51	chr14:1456098-1456737	90	GCCTGTCTCGCTACACTCA	CAAGAAGGGAGCAGGTGTT	61	640	415
52	chr14:4094578-4095072	90	TCTCCCTGGTGCTCTCTACA	GGTCATGGACTACTGAACTCCT	61	495	270
53	chr17:23773586-23774250	90	CAGATTCCTGCCTTTGTACCCT	TGTACGTCCCTGTGCTAACCAT	64	665	440
54	chr18:45818158-45818847	90	GATATGTTTGGGGAGATTCTGTC	CAATGGGAAAGGGCTATGAC	61	690	465
55	chr19:19379109-19379636	90	GCCCACAAGAGAGCTAACAG	ATATCAAACGGAGCCCAGGT	61	528	303
56	chr20:30137798-30138294	90	CCTCGTGGACAATACACTGAC	GACTTGGATGCAGGATGGT	61	497	272
57	chr23:28524731-28525286	90	CGCTGTAAGTGGGTCATGTGAG	ACACATGGCCAGCACCACTA	61	556	331
58	chr23:46986760-46987332	90	AGAGAGGAAGATAAGCTGAGGA	TTCTCTTGATCAGGATGCCA	60	573	348
59	chr26:12185278-12185852	90	CTTCCCTGAGCTGGACTCTG	CCTGGCTACATTCCTCATCCT	61	575	350
60	chr26:18131576-18132412	90	GCTACTAAAGTTGGCTCAGCATC	CCTCTCTCATTTCACCCACG	61	837	612
61	chr2:34656271-34656798	85	GGAGGTCGGTATTGGCAGAA	CACTTGCACGACCACTGTTCA	61	528	303
62	chr3:63101247-63102056	85	TGATCTATTTTGGTGGAGCAAGC	GCGCCAGCTGTTTCATGTAA	61	810	585

63	chr5:81797070-81797783	85	GCATTTTAATCTCAGACCACCTTG	TGATCCCCTAGGAATGCCTT	61	714	489
64	chr5:96841710-96842396	85	GACCTTGGAAGACAACTGCTA	ATCATGGCAGATATTTAAGTGTGA	60	687	462
65	chr6:11719974-11720356	85	ACAATCATACAAGCGCCTGTT	ACTTGGTTCCCTGCATGACA	61	383	158
66	chr6:42015096-42015962	85	AGGGGTGAGGTTACTGAGACA	AGCTGCTGCGGTATGTTGA	62	867	642
67	chr10:57161463-57161867	85	TTCAAAAGTCCCCAGGTTCT	ATCCTTGCCTTCCTTCCTGT	60	405	180
68	chr11:16281914-16282250	85	AACCAGACCGATGAGCAACA	TCTGCAGTTTGGTCCTGACCTA	61	337	112
69	chr11:16646510-16647064	85	GTTTGCCTTCTCCTTGCCTT	CTCATGGATTTGGGTGCAGT	60	555	330
70	chr11:26505572-26506324	85	CCGTCCTCTCCTGATCAGAAC	CTCTGAGGAGGCCAAACGA	61	753	528
71	chr15:4490166-4491122	85	AGCTGGCCTGCGAATGTAACTA	GTGTGAACCCTTTCCCAAGTGT	64	957	732
72	chr15:71625901-71626629	85	TGGAAGTGGTTGGATGCTCA	AGCTGTCTTTCATGGTCCTCTG	63	729	504
73	chr16:82384396-82385160	85	CCTGGGCATCTCATTAAGCA	GGCGTTAATACCATGGAGCAA	62	765	540
74	chr20:34809241-34809876	85	CTGATCTTATGTGAGTGTGCACC	AGCCCTCAGAAGACATCCTTG	61	636	411
75	chr21:18832793-18833523	85	GCTGAAACCCTGGACTATTGG	CATTGGGTGGCAGTACGTGT	61	731	506
76	chr22:6803196-6803968	85	ATGGCATAGGCACAAACAGC	GGGTCACTGGCTCAGAAAAGA	63	773	548
77	chr22:16854669-16855260	85	TAAGCCAGACTTCCGGAAGA	CCTGGTCAGTGCTGCTCA	60	592	367
78	chr23:6747870-6748508	85	GCAGTGGTTACCTGAGCAGAGT	CAGAGGAGGTGAAATAGGTTGG	62	639	414
79	chr27:26135013-26135389	85	AAGGGAAAGGGGCACAACT	TGGGCAGCAACTCGTTTCT	61	377	152
80	chr28:23074508-23075897	85	GAGAGGATGGATAAGCTGTGTTTAGG	GACTGACGCTGTAAATTAGAATGCC	62	1390	1161

Table S4B: primers used to clone the myostatin promoter and for reporter gene assay. The underlined nucleotides in primers MyostProm-F and MyostProm-R mark the *Hind*III and *Bam*HI restriction sites, respectively, used for cloning purpose.

Oligonucleotide name	Oligonucleotide sequence (5'-3')	Application
MyostProm-F	TACAAGCTTGAGTTGATAATTGGCAGCTACC	Cloning
MyostProm-F0	CAAATGAATCAGCTCACCCTT	Screening
MyostProm-R	ATAGGATCCTGAGAGACAACTTGCCACA	Cloning, screening
eGFP-F	TATCATGGCCGACAAGCAGAAGAAC	qPCR
eGFP-R	TTTGCTCAGGGCGGACTGGGTGCTC	qPCR
GAPDH-F	TCAACGACCACTTTGTCAAGC	qPCR
GAPDH-R	CCAGGGGTCTTACTCCTTGG	qPCR
humcavPRKC-RealT-F	CATCTGGAGTGAGCTGGACA	qPCR
cavPRKC-RealT-R	TGATTGGGATATGATGGAGCA	qPCR
